# Real-time understanding of lignocellulosic bioethanol fermentation by Raman spectroscopy

**DOI:** 10.1186/1754-6834-6-28

**Published:** 2013-02-20

**Authors:** Shannon M Ewanick, Wesley J Thompson, Brian J Marquardt, Renata Bura

**Affiliations:** 1 School of Environmental and Forest Sciences, University of Washington, Seattle, WA, USA; 2 Applied Physics Laboratory, University of Washington, Seattle, WA, USA

## Abstract

**Background:**

A substantial barrier to commercialization of lignocellulosic ethanol production is a lack of process specific sensors and associated control strategies that are essential for economic viability. Current sensors and analytical techniques require lengthy offline analysis or are easily fouled in situ. Raman spectroscopy has the potential to continuously monitor fermentation reactants and products, maximizing efficiency and allowing for improved process control.

**Results:**

In this paper we show that glucose and ethanol in a lignocellulosic fermentation can be accurately monitored by a 785 nm Raman spectroscopy instrument and novel immersion probe, even in the presence of an elevated background thought to be caused by lignin-derived compounds. Chemometric techniques were used to reduce the background before generating calibration models for glucose and ethanol concentration. The models show very good correlation between the real-time Raman spectra and the offline HPLC validation.

**Conclusions:**

Our results show that the changing ethanol and glucose concentrations during lignocellulosic fermentation processes can be monitored in real-time, allowing for optimization and control of large scale bioconversion processes.

## Background

The growing bioethanol industry produced 22.3 billion gallons of ethanol worldwide in 2011[1] and is replacing many non-renewable products with products derived from biomass. The cost to produce many of these products, however, is still not competitive with petroleum-derived counterparts. Processes to produce fuels and chemicals from petroleum have a wealth of online analytical sensors that permit them to operate at or near capacity with optimal process yields. This hyper efficiency is a necessary condition for profitability in producing high volume, narrow-profit margin products such as fuels and some commodity chemicals. The need for process efficiency – and hence the need for online sensors – is especially acute in biomass fed biorefineries due to the complexity and expense of the feedstock. Development of robust sensors for lignocellulosic biorefineries is as critical as the research that has gone into developing the processes themselves, but has received little or no attention. Process improvements (feedstock pretreatment, microorganisms, enzymes, etc.) will likely reduce costs in the future, but in both the short and long term, improving the efficiency of existing operations will have the greatest effect on overall process economics.

In a typical ethanol production process, raw biomass is first pretreated, then saccharified, fermented and purified. The liquid fraction following acidic pretreatment and saccharification is high in soluble lignin, phenolics, sugar degradation products (e.g. furfural) and monomeric and oligomeric hemicellulosic sugars. Monomeric sugars are fermented using microorganisms that primarily produce ethanol, so both high and low concentrations of sugar and ethanol must be monitored over the course of fermentation in order to ensure that the fermentation is proceeding optimally. Such a diverse mix of compounds can pose a challenge to current analytical methods; high performance liquid chromatography (HPLC) with refractive index detection is one of the only methods currently in use that can measure ethanol and carbohydrates simultaneously in the presence of the aforementioned compounds. Although capable of very high sensitivity, good separation and quantification of multiple component mixtures, sample preparation and analysis can be time consuming, costly and not suitable for a process environment. As such, HPLC is usually limited to offline analysis of samples, precluding its use for real-time, continuous analysis. Spectroscopic methods have the potential to rapidly and non-destructively analyze multiple components of a reaction mixture. Raman spectroscopy in particular is an established vibrational spectroscopy technique useful for determining both qualitative and quantitative molecular information from almost any type of sample (e.g. solid, liquid or gas) [2,3]. A Raman spectrum is obtained by exciting a sample with a laser and measuring the inelastic scattering of photons from the vibrations within the molecules. Raman spectroscopy has been used successfully to measure ethanol alone during fermentations [4-6], but these techniques have as yet not been fully utilized to provide on-line, real time measurements of lignocellulose-derived materials.

Our objective in this research was to evaluate the possibility of real-time, continuous lignocellulosic fermentation monitoring using Raman spectroscopy. We monitored the progress of fermentation of both a synthetic fermentation broth containing glucose and a steam-pretreated switchgrass hydrolysate in a controlled bioreactor using a novel Raman immersion probe inserted in a fast loop parallel sampling system. Chemometric analysis of the reactants and products was done using Principal Component Analysis (PCA) of the Raman spectra and a Partial Least Squares (PLS) model was developed and validated using HPLC data.

## Results

### Glucose fermentation

To first evaluate the effectiveness of the Raman probe under ideal fermentation conditions, a glucose solution was fermented using *Saccharomyces cerevisiae.* Although fermentation of glucose to ethanol is typically carried out as a batch process, a stepwise fed-batch experiment was run to follow the product consumption and formation rates more clearly over short periods of time. The experiment began with a glucose concentration of 5 g/L and additional aliquots of glucose were added at regular intervals when ethanol production had ceased to increase (determined by monitoring the intensity of the ethanol Raman peak at 883 cm^-1^ in real time) until a total of 25 g/L had been added. HPLC validation samples for determination of ethanol and glucose were withdrawn from the vessel at 10-15 minute intervals and Raman spectra were measured automatically every 30 seconds.

**Figure 1:**
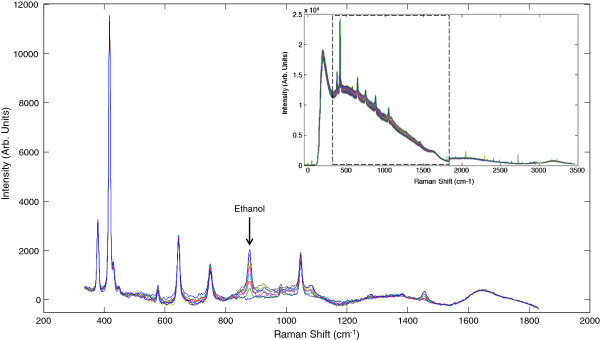
**Raw spectra (inset) were pretreated with a polynomial fitting routine to reduce the elevated background and a cosmic ray removal algorithm to remove spurious peaks caused by the high energy particles from the sun. **In the pretreated spectra, the ethanol peak can be easily seen at 883 cm^-1^.

Figure 1 (inset) shows the full Raman spectrum of the reaction mixture. The region from 350-1800 cm^-1^ shows an increased background due to water and some evidence of cosmic ray interference. To mitigate these effects and evaluate the elements of the spectra changing over time it was necessary to process the data with in-house data pretreatment algorithms. The background water spectrum removal algorithm is a modified polyfit algorithm [7], applying a moving window to the polynomial subtraction routine that reduces the effects of baseline shifts and fluctuating spectral background. Data were also processed using a cosmic ray removal filter [8] which compares each spectrum to the ones preceding and succeeding it, identifying the transient cosmic spikes and removing them. The remainder of Figure 1 shows the region of interest after all spectral pretreatment algorithms. These data were used for the development of multivariate models.

**Figure 2:**
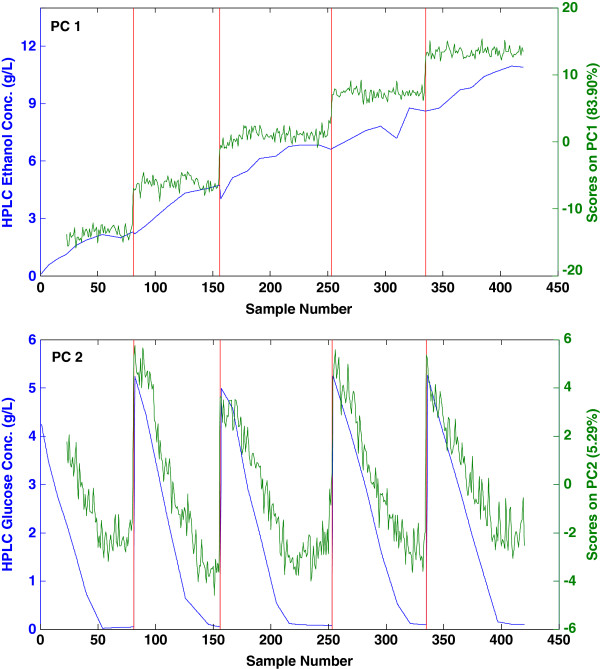
**There is a strong correlation of the scores data to the reference ethanol and glucose concentrations from HPLC. **The blue lines show the concentration of the analytes by HPLC while the green shows the principal component score of the Raman data. Ethanol correlates to PC1 (top) while glucose can be seen on PC2 (bottom). The red lines indicate added aliquots of glucose.

Principal Component Analysis (PCA) of the Raman spectroscopy data determined two components comprised 89% of the variance in the data and the scores of these components correlated well to the HPLC concentration data of ethanol on the first principal component (PC) and glucose on the second PC (Figure 2).

**Figure 3:**
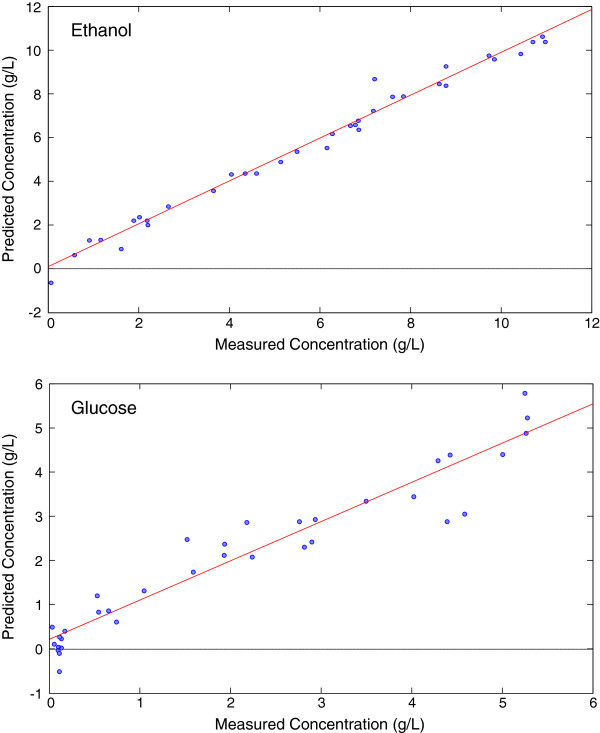
**Partial Least Squares models from the glucose fermentation. **Ethanol (top) and glucose (bottom) models were pretreated with orthogonal signal correction and cross validated using random subsets.

While simple monitoring of the fermentation rates provides some information, determining the actual concentration of the reactants is essential to compare the process to past and future processes. The spectroscopic data were evaluated by Partial Least Squares (PLS) using the HPLC results as the calibration concentration data set (Figure 3). The data were conditioned with Orthogonal Signal Correction (OSC) and mean centering to mitigate any non-relevant variation in the models. The PLS models were cross validated by removing random prediction subsets. Cross validation provides a means to evaluate the performance of the models by removing a subset of the data, generating a model from the remaining data and applying the subset as a test set. The Root Mean Square Error of Cross Validation (RMSECV) defines the model’s ability to accurately predict the test set samples. The models correlated well with the HPLC data over the full range of concentrations (0.1 – 11 g/L – ethanol and 0.1-5.5 g/L – glucose), and a standard limit of detection as defined as 2x the RMSECV allows quantification of 1 g/L and above for either ethanol or glucose (Table 1). These results indicate that even with the varied rates of glucose uptake and ethanol production, the reaction components were detected and followed over the course of the reaction.

**Table 1:** Prediction model data for both glucose and switchgrass hydrolysate fermentation models

		**R**^**2**^	**RMSEC**	**RMSECV**
**Glucose Fermentation**	**Ethanol**	**0.984**	**0.010942**	**0.40995**
	**Glucose**	**0.920**	**0.32228**	**0.5335**
**Hydrolysate Fermentation**	**Ethanol**	**0.935**	**0.2009**	**0.60326**
	**Glucose**	**0.513**	**0.20828**	**1.0614**

### Lignocellulosic hydrolysate fermentation

The initial experiments demonstrated that Raman spectroscopy could identify glucose and ethanol within the spectrum of a synthetic fermentation broth and monitor the process. The same sampling, spectral preprocessing, modelling and data analysis techniques were then applied to the fermentation of steam-exploded switchgrass hydrolysate; a dark brown solution produced by the reaction of switchgrass for a short time under high heat and pressure in the presence of SO_2_[9]. The hydrolysate is high in lignin and sugar degradation products, as well as monomeric and oligomeric carbohydrates from cellulose and hemicellulose. The hydrolysate was fermented in the same step-wise fashion as the synthetic fermentation. The concentration of monomeric glucose in the hydrolysate was relatively low (1.5 g/L), so additional glucose (3.5 g/L) was added at the beginning and 5 g/L of glucose added at regular intervals. Fermentation of each added amount of glucose proceeded until the glucose was consumed and ethanol production had ceased to increase as determined by monitoring the intensity of the ethanol Raman peak at 883 cm^-1^ in real time and verified by offline HPLC analysis.

**Figure 4:**
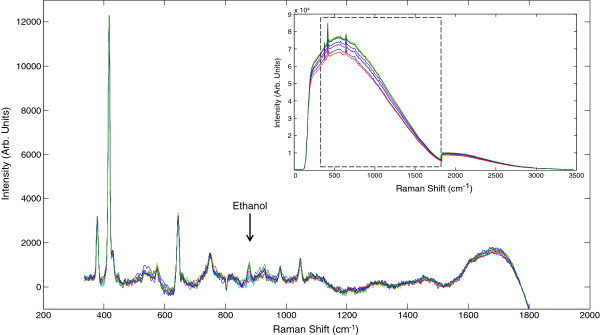
**Raw spectra from the hydrolysate fermentation (inset) were treated similarly to the synthetic fermentation data to remove the elevated background. **Noise is intensified in the pretreated spectra due to the greater intensity of the background signal and the heteroscedastic nature of the noise.

As expected, the switchgrass hydrolysate spectra exhibited a highly elevated spectral background compared to the synthetic fermentation broth, presumably due to the presence of fluorescent lignin-derived compounds (Figure 4, inset). The modified polyfit and cosmic ray removal algorithms greatly reduced the spectral background, illustrating that the spectra were reproducible and the ethanol peak at 883 cm^-1^ was readily apparent (Figure 4). Compared to the spectra of the synthetic broth, these spectra have visibly increased noise due to the heteroscedastic nature of the noise remaining after spectral pretreatment. Future work will focus on the reduction of background signal to improve our signal to noise ratio and modelling ability.

**Figure 5:**
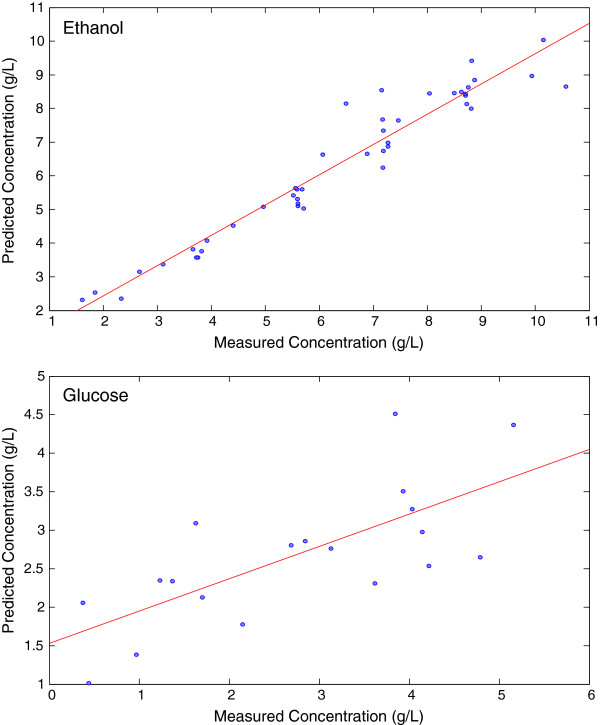
**Partial Least Squares models generated from hydrolysate fermentation data. **Ethanol (top) and glucose (bottom) data were pretreated similarly to previous models. For the glucose model, data below 0.03 g/L were assumed to be below the limit of detection and removed.

The hydrolysate fermentation spectra correlated well with the offline HPLC analysis for ethanol (Figure 5). The ethanol peak at 883 cm^-1^ is visually distinct from the baseline and models well even in the presence of an elevated spectral background, with a RMSECV only 0.2 g/L lower than the synthetic fermentation broth (Table 1). The concentration of glucose was more difficult to predict after the background pretreatment. The low concentration of glucose in our fermentation, combined with a high loading of yeast cells, yielded a glucose concentration that rapidly decreased culminating in calibration HPLC data below our current spectroscopic limit of detection (LOD). The synthetic fermentation glucose models had a limit of detection of about 1 g/L. Many of the calibration data for glucose in the hydrolysate fermentation fell below 0.03 g/L; removing these data provided a more robust model for glucose in the hydrolysate fermentation however this left only 19 reference points for modelling. Additional reference points may increase the robustness of the models; however, more important is reducing the spectral background signal in order to improve the signal-to-noise ratio and reduce prediction errors.

We have demonstrated quantitative models for following ethanol production during fermentation and while these models show promising results with a low concentration system, further study is necessary to alleviate the background effects that inhibit monitoring glucose consumption. The glucose concentration in a typical fermentation broth following hydrolysis of polymeric and oligomeric carbohydrates is 20-50 g/L, much higher than the starting glucose concentration in our stepwise fed batch hydrolysate fermentation. In the batch fermentation, the initial glucose was rapidly consumed resulting in low glucose concentrations during much of the fermentation. A realistic, large scale fermentation, however, would have a much larger range of concentrations of glucose which would improve the results from our real time analysis.

## Discussion

Despite widespread use in other areas, Raman spectroscopy has not yet been utilized extensively for continuous analysis of fermentation of lignocellulosic-derived materials. However, small scale ethanol fermentations have been measured by Raman spectroscopy, both through periodic removal and measurement of samples [10] and continuous monitoring of nano-scale reactions with in situ measurement [5]. In addition, Shih et al [11,12] used a 785 nm Raman microscope to measure offline aliquots of both ethanol and sugar from enzymatic hydrolysis and fermentation of pretreated corn stover. Multiple sugars and ethanol were measured simultaneously, but high background fluorescence in the spectra was problematic. Attempts to decrease the spectral background and increase the detection limits by extraction of the biomass prior to sample pretreatment with solvents (ethanol, hexane or water) were successful in reducing the LOD for glucose from 20 g/L to 4 g/L, but such treatments are impractical on a larger scale [11]. An elevated spectral background signal is a persistent issue when dealing with lignocellulosic biomass fermentation processes – lignin is made up of highly conjugated phenolic groups [13], which can lead to an elevated background signal in the same spectral region as the compounds of interest, potentially masking the Raman features of the spectra [14].

Analysis of ethanol and glucose has also been conducted non-spectroscopically in a number of ways in order to eliminate the need for manual sampling and the associated delay in data procurement. Sequential injection analysis (SIA) with enzyme or amperometric detection [15,16] can measure both ethanol and glucose in solution. Indirect monitoring of fermentation progress by measurement of headspace CO_2_[17] and electrochemical detection of ethanol by microelectrode array [18] provide information about the progress of the reaction, but cannot pinpoint a cause if the reaction deviates from normal conditions. These methods are an improvement over offline HPLC methods, but still cannot provide information in real time.

The advantages of online Raman spectroscopy over other methods lie mainly in the speed of analysis and the reduction of user interaction. The Raman spectroscopy method was capable of collecting a full spectrum every 30 seconds, from which both ethanol and glucose concentrations could be determined within seconds using multivariate control models. Following the quantities of ethanol and glucose in near real-time provides insight regarding fermentation performance and allows for control decisions to be made in time to affect the quality of the product being formed in the bioprocess.

Many reactions are run for a set period of time depending on their initial sugar concentration, yeast loading, temperature, etc. These parameters are determined based on theoretical values and previous experiments. However, other factors may influence the fermentation rate and so often fermentation processes are run longer than necessary to ensure completion. Raman spectroscopy allows the user to determine if the cell loading was sufficient, detect possible contamination, determine the rate of fermentation and see exactly when the fermentation has completed. In addition, process upsets or problems can be spotted early, reducing costs and increasing efficiency.

## Conclusions

Fermentation of both a synthetic fermentation broth and a lignocellulosic hydrolysate was measured continuously by 785 nm Raman spectroscopy. Despite an elevated background present in the lignocellulosic hydrolysate, effective data pretreatment methods allowed for measurement of ethanol and glucose over the course of the reaction. These results show that Raman spectroscopy has the potential to be an effective tool to improve the efficiency of existing bioconversion processes. With precision sensors continuously monitoring large scale reactions, time and resources can be conserved to help ensure economic sustainability of biomass-based biorefineries in the long term.

## Methods

### Steam-pretreated switchgrass hydrolysate

The liquid hydrolysate was prepared as described by Ewanick and Bura [9]. Briefly, four 50 g aliquots of SO_2_-impregnated switchgrass were sequentially steam-exploded using a 1.5 L batch steam gun (HM^3^ Energy Inc, Gresham OR) at 195°C for 7.5 minutes. The resulting slurry, at 14% consistency, was separated by vacuum filtration into a liquid fraction (used in this study) and solid fraction and stored at 4°C.

### Fermentation

*Saccharomyces cerevisiae* ATCC 96581 isolated from spent sulphite liquor [19] (obtained from ATCC) was streaked onto YPD agar plates and allowed to grow for 48 hours. Prior to fermentation, pre-culture cells were grown by adding one colony from the plate to liquid media containing 10 g/L each of glucose, yeast extract and peptone. After 24 hours of growth at 30°C and 150 rpm orbital shaking, the cells were centrifuged and the spent supernatant removed and replaced with fresh media. The cells were then grown for another 24 hours under the same conditions; the cells were again spun down, washed twice in deionized water, and then resuspended in a small volume of 0.9% sodium chloride. Cell concentration was determined by measuring the optical density of the suspension at 600 nm and comparing to a calibration curve prepared using oven dried cells at varying optical densities.

Synthetic fermentation broth solutions, as well as the steam-pretreated hydrolysate were adjusted to pH 6 using dilute NaOH. Nutrients in the form of ammonium phosphate (2 g/L), sodium sulfate (0.2 g/L) and sodium nitrate (2 g/L) were added and the solution was heated to 30°C in a 1.3 L New Brunswick Scientific BioFlo 115 bioreactor equipped with a water jacket, exhaust condenser and pH probe. The pH was monitored and maintained at pH 6 for the duration of the fermentation with 1 M HCl and 2 M NaOH. The total solution volume was 800 mL with a cell concentration of 5 g/L and the mixture was stirred continuously with a Rushton impellor at 400 rpm. The initial glucose concentration was 5 g/L at time zero, and further 4 g aliquots of glucose were added when the Raman ethanol peak at 883 cm^-1^ reached equilibrium, roughly every 90 minutes. One milliliter samples were removed every 10-15 minutes for HPLC analysis.

### Raman data collection and analysis

Real-time analysis data were collected using a RamanRxn1 instrument (Kaiser Optical Systems, Ann Arbor, MI). The excitation wavelength was 785nm with a power at the sample of 250mW. Spectra were collected as an average of six, five-second exposures resulting in a collection time of 30 seconds per spectrum. A ballprobe immersion optic (Matrix Solutions, WA) was used for collection of the spectroscopic data. The spherical lens of the ballprobe collects the signal from a small volume very close to the ball surface, providing a constant focal length and greatly enhanced measurement precision. The spherical tip of the probe causes high shear forces as the reaction liquid circulates in the sampling system, preventing accumulation of cells or debris on the probe surface.

A custom sampling loop system that rapidly pumped the fermentation broth out of the fermenter, past the probe and back into the vessel was used with the ballprobe to reduce the possibility of fouling and improve the sampling reproducibility of the fermentation. The slightly increased pressure generated in the sampling loop maintains gases in solution, thus preventing CO_2_ bubbles produced during fermentation from interacting with the excitation laser and potentially causing erroneous data points. The sampling loop was designed using NeSSI (New Sampling/Sensor Initiative) sampling blocks that provide a simplified flow path past the ballprobe. NeSSI defines a standard physical format (ANSI/ISA SP76.00.02) to simplify development and installation, and reduce the size of fluid handling systems. The fast loop system was developed using Parker Intraflow (Cleveland, OH) substrates and top mount components and had a volume of approximately 10 ml. The fermentation broth was pumped through the fast loop at 500 mL/min to ensure a rapid sample turnover in the fermenter.

### HPLC analysis

Ethanol and glucose were measured using refractive index detection on a Shimadzu Prominence LC. Samples were diluted as appropriate, filtered through 0.22 μm syringe filters and 20 μL of sample were injected onto a Phenomenex Rezex RHM H^+^ column at 63°C with an isocratic mobile phase elution of 0.05 mM H_2_SO_4_ at 0.6 ml/min. Standards were prepared and used to quantify the unknown samples.

### Data analysis

Data models were created and analyzed using Matlab (TheMathWorks, MA) and the PLS_Toolbox (Eigenvector Research, Inc., WA).

## Competing interests

The authors declare that they have no competing interests.

## Authors’ contributions

SME performed fermentation and data analysis and participated in drafting the manuscript. WJT performed Raman data collection and analysis and participated in drafting the manuscript. RB and BJM conceived experiments and participated in drafting the manuscript. All authors read and approved the final manuscript.
